# Network analyses: Inhibition of androgen receptor signaling reduces inflammation in the lung through AR-MAF-IL6 signaling axes

**DOI:** 10.1016/j.gendis.2023.07.001

**Published:** 2023-08-18

**Authors:** Albert R. Wang, Andrew M. Baschnagel, Zijian Ni, Sean R. Brennan, Hypatia K. Newton, Darya Buehler, Christina Kendziorski, Randall J. Kimple, Gopal Iyer

**Affiliations:** a Department of Human Oncology, University of Wisconsin School of Medicine and Public Health, University of Wisconsin, Madison, WI 53705, USA; b Department of Biomedical Engineering, University of Wisconsin-Madison, Madison, WI 53705, USA; c University of Wisconsin Carbone Cancer Center, Madison, WI 53705, USA; d Department of Biostatistics and Medical Informatics, University of Wisconsin-Madison, Madison, WI 53706, USA; e Department of Biology, Tufts University, Medford, MA 02155, USA; f Department of Pathology and Laboratory Medicine, University of Wisconsin School of Medicine and Public Health, University of Wisconsin, Madison, WI 53705, USA

Androgen receptor (AR) is a major transcription factor that plays a role in inflammatory response including interleukin-6 (IL6) signaling.^1^ While AR regulation through paracrine loop signaling in prostate tissue is well-studied, its impact through an IL6 autocrine loop in the lung has not been well-studied despite the organ's response to respiratory viral infection. Chemical inhibition and RNA knockdown of AR identified a bZIP transcription factor MAF to be a common target of inflammation using these perturbations in lung cells. We hypothesized through a predictive *in silico* network modeling that MAF is a common mediator between androgen signaling and inflammatory response in lung cells exposed to AR antagonists and viral (SARS-Cov-2) infection.

To establish a context for AR inhibition in the lung, a public database of 427 normal human lung samples revealed AR expression with levels not significantly varying by gender or age (Fig. S1A). This suggested that AR is expressed in normal lungs and is not unique to prostate tissue. A subset of six lung cell lines characterized with AR antagonist enzalutamide for their EC_50_ (Table S1) was treated for 0.5, 2, 4, 8, or 24 h in the presence of AR agonist R1881 (testosterone), which revealed AR expression in A549 cells to be negatively feed-back regulated (Fig. 1A) while H226, H460, H2228, and H3122 cells were minimally responsive up to 24 h, with the exception being H520, a typical squamous lung cell line (Fig. S1B). We chose to further characterize AR early (2 h) and late (24 h) expression in A549 and H2228 cells. While enzalutamide actively displaced the agonist R1881 and vice versa and induced AR down-regulation as early as 2 h up to 24 h, enzalutamide treatment in the absence of R1881 was similar to control treatment in A549 cells, suggesting a reversible on-off antagonist-agonist interaction up to 24 h. However, in H2228 cells, similar treatments induced a slight increase in AR expression (Fig. 1B). Unlike prostate cells which are negatively regulated by R1881, only bronchioalveolar cells (A549), a cell-type progenitor for inflammation and carcinoma, showed sustained AR signaling in controlled charcoal fetal bovine serum stripped of hormone treatments. Next, we established that AR was functional in the lung through dynamic nuclear-cytoplasmic imaging and quantitation. While AR (red) was efficiently transported to the nucleus in both A549 and H2228 cells with ligand R1881 (Fig. 1C; Fig. S2A), an effect that was disrupted using either AR-specific siRNAs (Fig. S3 and Table S2) or enzalutamide and quantified (Fig. 1D; Fig. S2B), demonstrating that siRNA knockdown of AR phenocopies the effect of enzalutamide.

**Figure 1:**
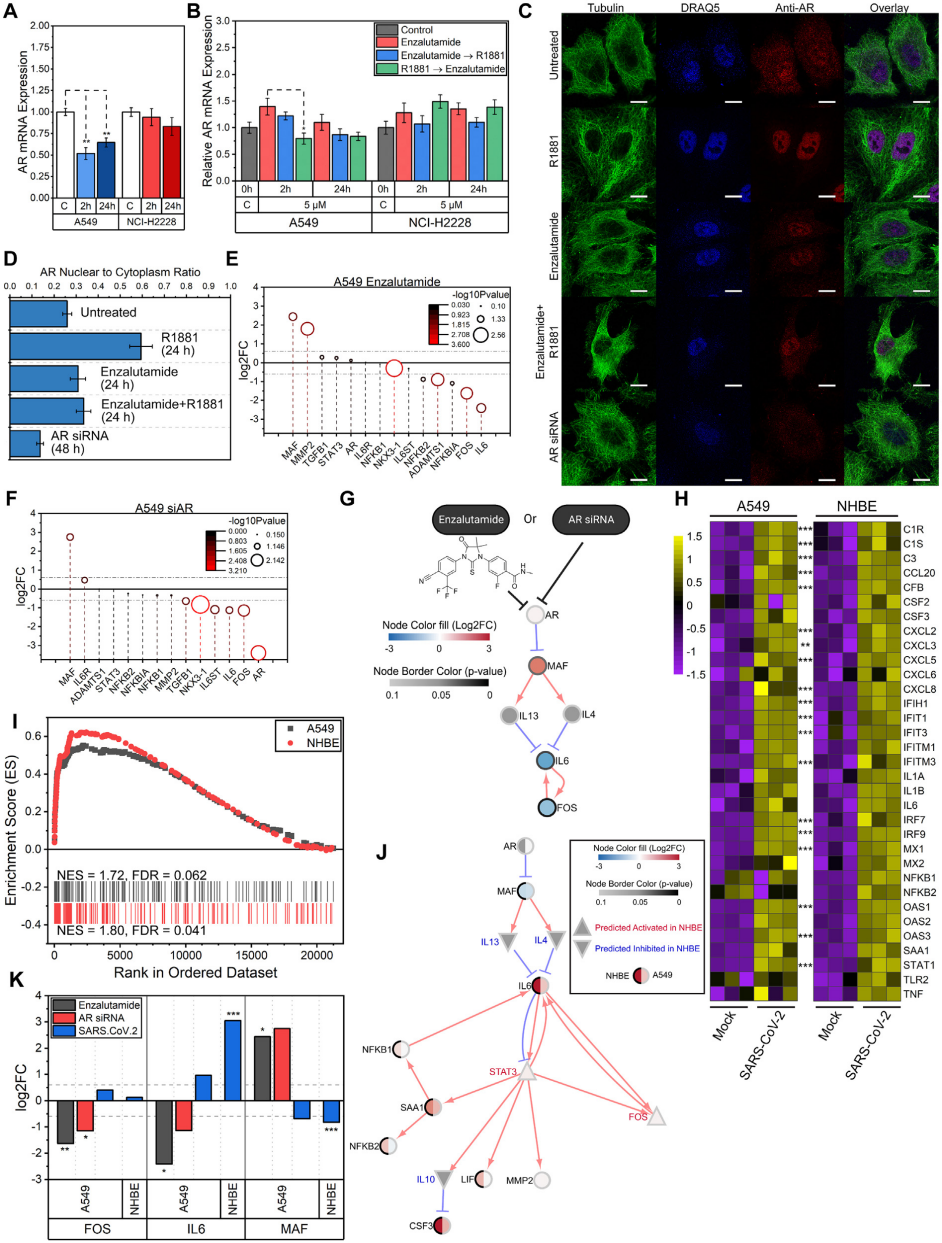
Androgen receptor inhibition converges to predict an inflammatory transcriptional network in the lung. **(A)** Fold change (FC) of AR after 1 nM R1881 stimulation for 2 h and 24 h mRNA expression of treated samples was normalized to vehicle control (white bar), and the significance was determined using a two-sample *t*-test (^∗^*P* < 0.05, ^∗∗^*P* < 0.01, ^∗∗∗^*P* < 0.001). The data were presented as FC ± standard error. **(B)** Comparison of AR mRNA levels in three conditions of enzalutamide treatment: enzalutamide alone (red), pre-treated with enzalutamide for 30 min prior to induction of 1 nM R1881 (blue), and pre-treated with R1881 for 30 min prior to enzalutamide treatment (green). **(C)** Representative immunofluorescent images of AR protein in A549 cells treated with 1 nM R1881, 5 μM enzalutamide (with or without 1 nM R1881), or AR siRNA (scale bar = 10 μm). **(D)** The ratio of quantified AR immunofluorescent signals within the nucleus to the entire cell region (A549). The data were presented as mean ± standard deviation. **(E, F)** Log_2_ fold change (log_2_FC) gene expression profile of enzalutamide- and siRNA AR-treated A549 cells. Genes with absolute values of log_2_FC greater than 0.6 (represented by horizontal grey dash-dotted lines) and *p* value less than 0.05 (or 1.3 in −log 10 scale) are considered significant. Circles with larger diameters and colored red represent having more significant *p* values. **(G)** Respective DEGs of enzalutamide- and AR siRNA-treated A549 cells were used to construct separate gene interaction networks. Relationship between AR and IL6 is established through MAF, IL4, and IL13. Node color represents log_2_FC (red: up-regulation, blue: down-regulation, grey: not measured), while the border of the node is darker as the *p* value becomes more significant. Any isolated node was hidden in the final diagram. **(H)** Inflammatory gene expression of SARS-CoV-2 infected A549 and NHBE. All genes are differentially expressed for NHBE, while the DEGs for A549 are marked with asterisks (adjusted *P* value: ^∗∗^*P* < 0.01, ^∗∗∗^*P* < 0.001). Colors represent z-scores of the normalized read counts across all samples within individual cell lines. **(I)** Inflammatory response (M5932) was one of the top significant gene sets when GSEA analysis was performed on MSigDB's Hallmark gene sets (H collection, v7.1). The respective normalized enrichment scores (NES) and false discovery rates (FDR) are shown for A549 (top) and NHBE (bottom). **(J)** Using the DEGs of the SARS-CoV-2 infected cells, a gene interaction network was constructed based on the JAK-STAT signaling pathway (KEGG database) and genes of interest (see methods for more details). Gene expression from NHBE and A549 sequencing data was plotted side by side (left half circle: NHBE; right half circle: A549) for comparison. Compared with AR inhibitory treatment in A549 (Figure 1G), viral infection causes opposite gene expression of IL6 and MAF in NHBE/A549. **(K)** Comparison of gene expressions (log2FC) obtained from the lung cells treated with enzalutamide (dark gray), AR siRNA (red), or SARS-CoV-2 (blue).

To identify downstream AR-inhibited signaling targets, gene expression analyses were performed on A549 and H2228 cells treated with enzalutamide (5 μM) for 24 h or AR siRNA (10 nM) for 48 h. Notably, significant up-regulation of bZIP transcription factor (MAF) (enzalutamide: log_2_FC = 2.44, *P* = 0.035; AR siRNA: log_2_FC = 2.75, *P* = 0.056) and down-regulation of IL6 (log_2_FC = −2.41, *P* = 0.023; log_2_FC = −1.13, *P* = 0.0502) and FOS (log_2_FC = −1.627, *P* = 0.008; log_2_FC = −1.15, *P* = 0.0102) among other downstream targets were observed in A549 cells following enzalutamide treatment (Fig. 1E) and AR siRNA treatment respectively (Fig. 1F). In contrast, H2228 cells demonstrated up-regulation of IL6 and FOS, while MAF decreased following AR blockade (Fig. S4A, 4B). The increased expression of IL6 in H2228 cells resembles a suggested androgen-independent disease progression mechanism in prostate cancer and induces some AR target genes through activation of signal transducer and activator of transcription 3 (STAT3).^2^

The contrasting expression profiles of A549 and H2228 potentially attributed to androgen-independent pathways through IL6 signaling led us to build a predictive regulatory network from all treatments, specific to A549 cells. Using two types of data variables (differentially expressed genes (DEG) with *p* value < 0.05 and absolute log_2_FC ≥ 0.6; network of regulatory interactions calculated by the impact analysis method^3^), we identified DEGs as nodes with the edges representing regulatory interactions between two genes for each treatment. The network deploys only edges observed in STRING and BioGRID databases with a high confidence of ≥700. The network predicted that blocking AR signaling with enzalutamide and siRNA converge towards MAF up-regulation due to the removal of AR inhibitory interaction on MAF which results in decreased expression of IL6 in part through IL13 and IL4 (Figure 1G). With MAF as a node with edges to predict IL4 and IL13 to have inhibitory effects on IL6 resulting in suppression of IL6 expression (Fig. 1G and Table S3), we asked if this network would have an opposite regulatory interaction when IL6 was up-regulated.

We chose SARS-CoV-2 viral infected gene expression datasets from A549 and NHBE lung cells since IL6 expression was up-regulated post-infection.^4^ DEGs in A549 and NHBE also have similar inflammatory gene expression patterns and Gene Ontology (GO) terms (Fig. 1H and Table S4). Gene set enrichment analysis (GSEA) revealed inflammatory response to be one of the top enriched gene sets found in both A549 and NHBE (Fig. 1I). Hallmark gene sets such as IL6-JAK-STAT3 signaling, complement, TNFA signaling via NF-kB, and interferon responses were also highly enriched in both cell lines (Table S5). Notably, similar transcriptional profiles were observed between NHBE and A549 cells, and the network analysis revealed the same regulatory interactions between AR and IL6 (Fig. 1J; Fig. S5A and Table S6) as those present in the AR data treated with enzalutamide or AR siRNA (Fig. 1G). In contrast to the gene expression data from A549 following AR enzalutamide or siRNA treatment, viral infection induced down-regulation of MAF with up-regulation of IL6 (Fig. 1K). In A549 cells with a viral infection, IL6 was up-regulated (log_2_FC = 0.968) and MAF was down-regulated (log_2_FC = −0.68); opposite regulation in AR-enzalutamide and siRNA-inhibited A549 cells was also found, suggesting that MAF could be a co-regulatory partner with AR independently in the induction of IL6 expression.

Further, according to an additional predictive network using the upstream regulator analysis, IL6 activated STAT3 and subsequently serum amyloid A1 (SAA1) which is known to activate NF-κB signaling in lung cells (Table S3). NFKB1 could be involved in the activation of IL6, thus forming a positive autocrine feedback loop between IL6, STAT3, SAA1, and NFKB1^5^ (Fig. 1J). Moreover, IL6 and STAT3 are the top two nodes in terms of degree centrality and betweenness centrality (Fig. S5B and Table S6), indicating that they are the most connected nodes and the crucial gatekeepers in this network, which contains many genes related to inflammatory response and/or from the JAK-STAT pathway (Fig. S5A).

The predictive network analyses provide the basis for future co-culture experiments with immune cell types to elicit the combinatorial role of IL4-IL13-IL6 toward inducing inflammation. Further, these network analyses need to be experimentally validated through knockdown of MAF to reveal its role as a hub gene in regulating IL4-IL13-IL6 connectivity to show the significance of the pathway's protective role against the virus and modulating inflammatory pathways. Therefore, taking into consideration of our findings, we propose parallel *in silico* networks of AR inhibition mediated through enzalutamide to dampen the inflammatory response through IL6 as predicted through the regulatory connections between the AR-MAF-IL4-IL13-IL6 signaling and canonical IL6-STAT3-NFKB1 activation in lung cells that can shape the autocrine loop feedback (Fig. 1J).

## Ethics declaration

The study did not involve human subjects. All experiments were approved and are compliant by the Office of the Vice Chancellor for Research and Graduate Education Biosafety institutional committee at the University of Wisconsin-Madison Biosafety.

## Conflict of interests

The authors declare no potential conflict of interests.

## Funding

This study was funded in part by Astellas-Pfizer and was supported by the University of Wisconsin School of Medicine and Public Health and the University of Wisconsin Carbone Cancer Center Support Grant P30 CA014520 and UW School of Medicine and Public Health (SMPH) and UWCCC grant to Gopal Iyer. All lung cell lines except NCI-H3122 were received as research support as part of the ATCC Innovation Challenge.
