# Supplementary material for: Network analyses: Inhibition of androgen receptor signaling reduces inflammation in the lung through AR-MAF-IL6 signaling axes
Source: Genes Dis. 2023 Aug 18;11(3):101072. doi: 10.1016/j.gendis.2023.07.001 (PMC10825295; doi:10.1016/j.gendis.2023.07.001)
Supplement: Multimedia component 1 [file mmc1.docx]

**Supplementary data for**

**Network analyses: Inhibition of androgen receptor signaling reduces inflammation in the lung through AR-MAF-IL6 signaling axes**

Albert R. Wang^1,2^, Andrew M. Baschnagel^1,3^, Zijian Ni^4^, Sean R. Brennan^1,5^, Hypatia K. Newton^3^, Darya Buehler^3,6^, Christina Kendziorski^4^, Randall J. Kimple^1,3^, Gopal Iyer^1,3*^

1. Department of Human Oncology, University of Wisconsin School of Medicine and Public Health, University of Wisconsin, Madison, WI, 53705, USA
2. Department of Biomedical Engineering, University of Wisconsin-Madison, Madison, WI, 53705, USA
3. University of Wisconsin Carbone Cancer Center, Madison, 53705, WI USA
4. Department of Biostatistics and Medical Informatics, University of Wisconsin-Madison, Madison, WI, 53706, USA
5. Department of Biology, Tufts University, Medford, MA, 02155, 53705,

USA

1. Department of Pathology and Laboratory Medicine, University of Wisconsin School of Medicine and Public Health, University of Wisconsin, Madison, WI, USA

* Corresponding author: Gopal Iyer, PhD

Email: giyer@humonc.wisc.edu

**Authors’ contributions:** A.R.W. and G.I. designed the study. A.R.W and S.R.B. performed experiments using lung cancer cell lines. A.R.W. and S.R.B. performed analysis of gene expression data and immunofluorescence confocal images. Z.N. and H.K.N. performed statistical analysis of the RNA-seq data. A.R.W. performed GSEA, GO, and network analysis. A.R.W and G.I. prepared the manuscript with critical reading and input from all co-authors.

**This file includes:**

Methods and Materials

Supplementary Fig. S1 to S5

Supplementary Table S1 to S6

## Materials and methods

### AR gene expression in normal lung

Normalized transcript data from 427 lung tissues was extracted from the Human Protein Atlas (<http://www.proteinatlas.org>). Data originated from the Genotype-Tissue Expression project (<https://gtexportal.org>). RNA-seq data from lung tissues was mapped based on RSEMv1.2.22 (v7) and the resulting transcripts per million values were normalized as described (<https://www.proteinatlas.org/about/assays+annotation>).

### Cell culture

Lung cell lines, ATCC^®^ CCL-185™ (A549), CCL-5935™ (NCI-H2228), CCL-5826™ (NCI-H226), HTB-177™ (NCI-H460), and HTB-182™ (NCI-H520), were received as research support as part of the ATCC Innovation Challenge. All cell lines were cultured in RPMI 1640 media with 5% (v/v) charcoal-stripped fetal bovine serum and maintained at 37 °C with 5% CO_2_. Cells were treated with either 1nM R1881 (Metribolone, Sigma), 5µM enzalutamide, or combinations of both (pre-treated with enzalutamide for 30 min prior to induction of R1881. For AR siRNA, three different siRNA sequences (siAR1 to 3) were designed to target AR mRNA (IDT). Based on the knockdown efficiency of each sequence, the combination of siAR2 and siAR3 (50% each, at 10nM concentration and 48h transfection time) using Lipofectamine RNAiMAX (Thermo), with scrambled siRNA as control was used for all the siRNA treatments.

Cell viability for enzalutamide treatments at concentrations (0.5 to 200µM) for 72h was determined by using PrestoBlue reagent (Thermo). Relative fluorescence units (RFU) were measured with CLARIOstar Plus microplate reader (BMG Labtech). The raw absorbance values were first subtracted by the averaged absorbance value of background control (medium only). The cell survival rate at each concentration was calculated by normalizing to the signal of vehicle control samples. The data was curve fitted using the sigmoidal dose-response equation (Y= Bottom + (Top-Bottom)/(1+10^((LogIC50-X)*HillSlope)) in OriginLab to determine the EC_50_ values.

### Gene Expression

RNA samples collected with TRIzol (Invitrogen) were isolated using RNeasy Mini Kit (Qiagen) from three independent biological experiments for qRT-PCR. The fold changes and p-values were calculated with the ∆∆Ct method and two-sample t-test, respectively. Ubiquitin C (UBC) was used as the housekeeping gene for normalization.

AR: AGTACTGAATGACAGCCATCTG; CAACAACCAGCCCGACT

UBC: CCTTATCTTGGATCTTTGCCTTG; GATTTGGGTCGCAGTTCTTG

AR signaling pathway were screened using Bio-Rad’s PrimePCR 384-well gene panel. Bubble plots of Log_2_-transformed expression levels and -log10(p-value) of all treatment groups were graphed in OriginLab.

### Imaging with quantification

Lung cells were cultured on coverslips (#1.5), treated with R1881, enzalutamide and combination of enzalutamide/R1881 for 24h, and AR siRNA for 48h and treated with primary antibodies: Anti-AR rabbit (Cell Signaling Technologies); Anti-α-Tubulin mouse (Sigma-Aldrich) at 1:500 dilution and stained with 1:500 dilution of Donkey anti-rabbit Alexa Fluor 594 and Goat anti-mouse Alexa Fluor 488 and nuclear counter-stained with DRAQ5. Imaging was performed on Leica SP8 scanning confocal microscopy using 63X oil immersion objective. Multiple images of different fields were obtained for at least 100 cells for AR quantification. The sum of pixel intensities of AR signal within the nucleus and cytoplasm was quantified using a custom MATLAB program. Tubulin and DRAQ5 channels were used to create masks for the cytoplasm and nucleus regions, and then used to determine the AR nucleus-to-cytoplasm ratio.

#### Statistical Analyses

Raw sequencing data of SARS-CoV-2 treated and control samples from A549 and NHBE cell lines in Blanco-Melo *et al.* study were downloaded from GEO (GEO accession: GSE147507). A reference genome was manually built by combining GRCh38.p13 from RefSeq with SARS-CoV-2 (GenBank accession: NC_045512.2), respiratory syncytial virus (GenBank accession: NC_001803.1), and influenza A virus (GenBank accession: AF389115.1-AF389122.1) genomes. Quality control of single-end sequencing files was conducted using FastQC v0.11.7. Reads were aligned using STAR v2.7.1a (Dobin et al., 2012). The gene-by-sample count matrix was calculated using RSEM v1.3.0. Genes with average expressions less than 1 were filtered out. Differential expression (DE) analyses were performed between SARS-CoV-2 treated and control samples from A549 and NHBE separately using DESeq2 v1.24.0 under R v4.0.0. P-values were adjusted via Benjamini-Hochberg for multiple tests. Those genes with adjusted p-values less than or equal to 0.05 and absolute values of log_2_ fold change greater or equal to 0.6 were selected as significant DE genes.

### RNA-sequencing with network analysis

GSEA was used to perform enrichment analysis on the A549 and NHBE RNA-seq using Hallmark gene sets, and false discovery rate (FDR) less than 0.25 were considered enriched. The network analysis for AR signaling gene expression and the RNA-seq data sets from the Blanco-Melo et al. were done by using Advaita Bio’s iPathwayGuide (version 1910). Network for each treatment condition was constructed by including all differentially expressed genes (DEGs) as input nodes. Integrating one intermediate gene between any two input nodes (genes) was allowed so that we could further explore the relationships of these genes. In addition, some gene nodes were manually added for easier comparison between enzalutamide (AR) and AR siRNA (IL6 and MAF) treated cells. From COVID-19 data sets, the DEGs of NHBE cells from the JAK-STAT signaling pathway (KEGG) along with genes of interest (IL6, IL6R, IL6ST, AR, FOS, STAT3, ADAMTS1, ADAMTS17, TGFB1, MAF, NKX3-1, MMP2, IGFBP5, SOCS3, NFKB1, NFKB2) were used to build the gene interaction networks. Only regulatory interactions (activation and inhibition) and interactions with high confidence (score > 700) were considered in this study. The predictions for activated or inhibited genes were calculated based on the interactions with their downstream DEGs, and the p-values were adjusted via the Benjamini-Hochberg method to control the FDR. Gene Ontology (GO) term results were also generated by iPathwayGuide. The p-values of GO terms were corrected using the Elim pruning method, which emphasizes the most specific term first.

### Data and code availability

RNA-seq data from published study were downloaded from GEO (GEO accession: GSE147507). Other data and codes that support the findings of this study are available from the corresponding author upon reasonable request.

##### A


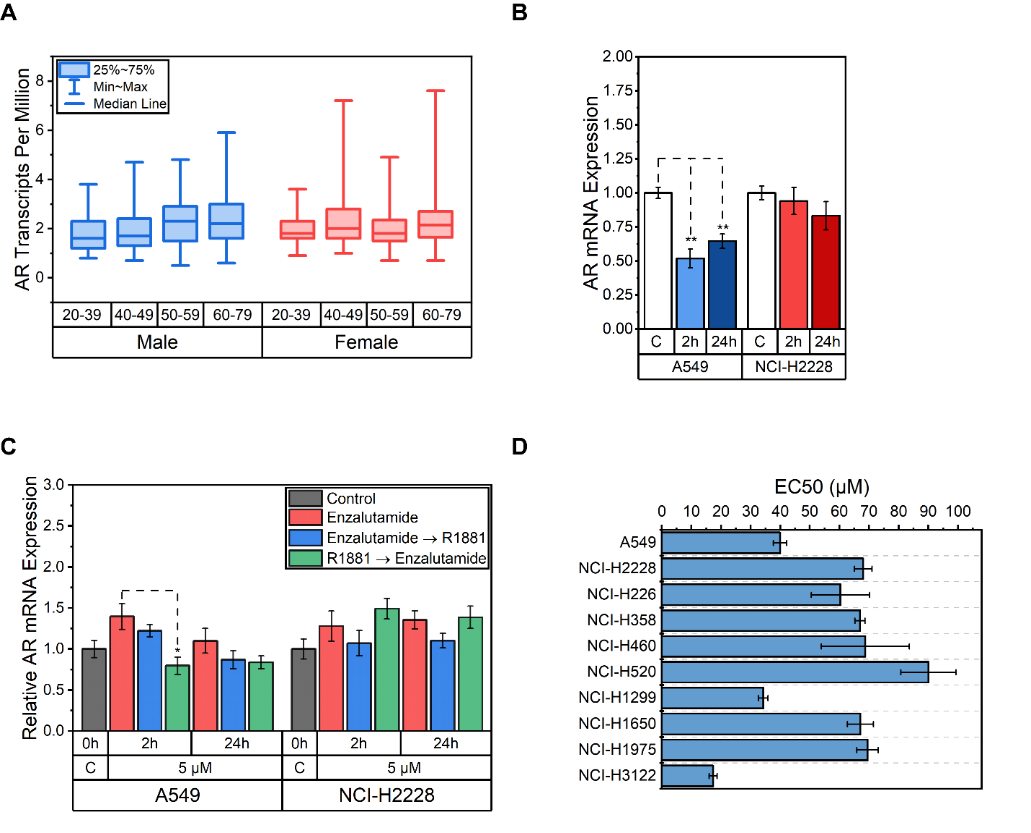


**B**


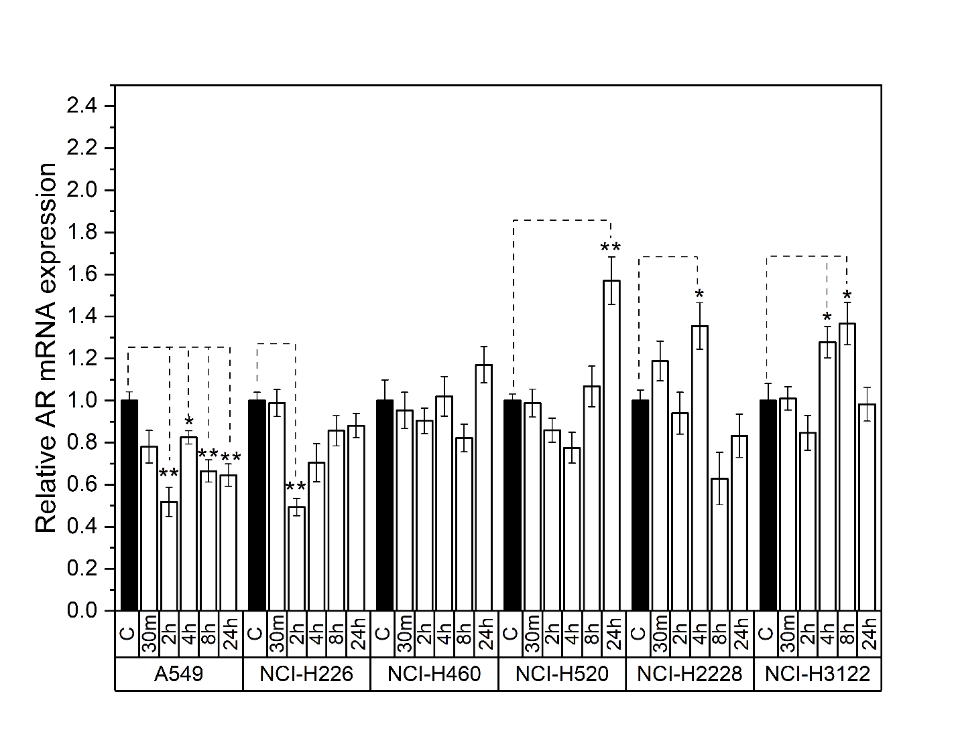


**Supplementary Figure S1 (A)** AR mRNA expression from 427 normal lung samples at different ages and genders. **(B)** Time course analyses of AR gene expression in lung cell lines (n=6) treated with 1 nM R1881. mRNA expressions of treated samples were normalized to vehicle control (black bar), and the significances were determined using two-sample t-test (*P<0.05, **P<0.01, ***P<0.001). Data is presented as fold change ± SE.


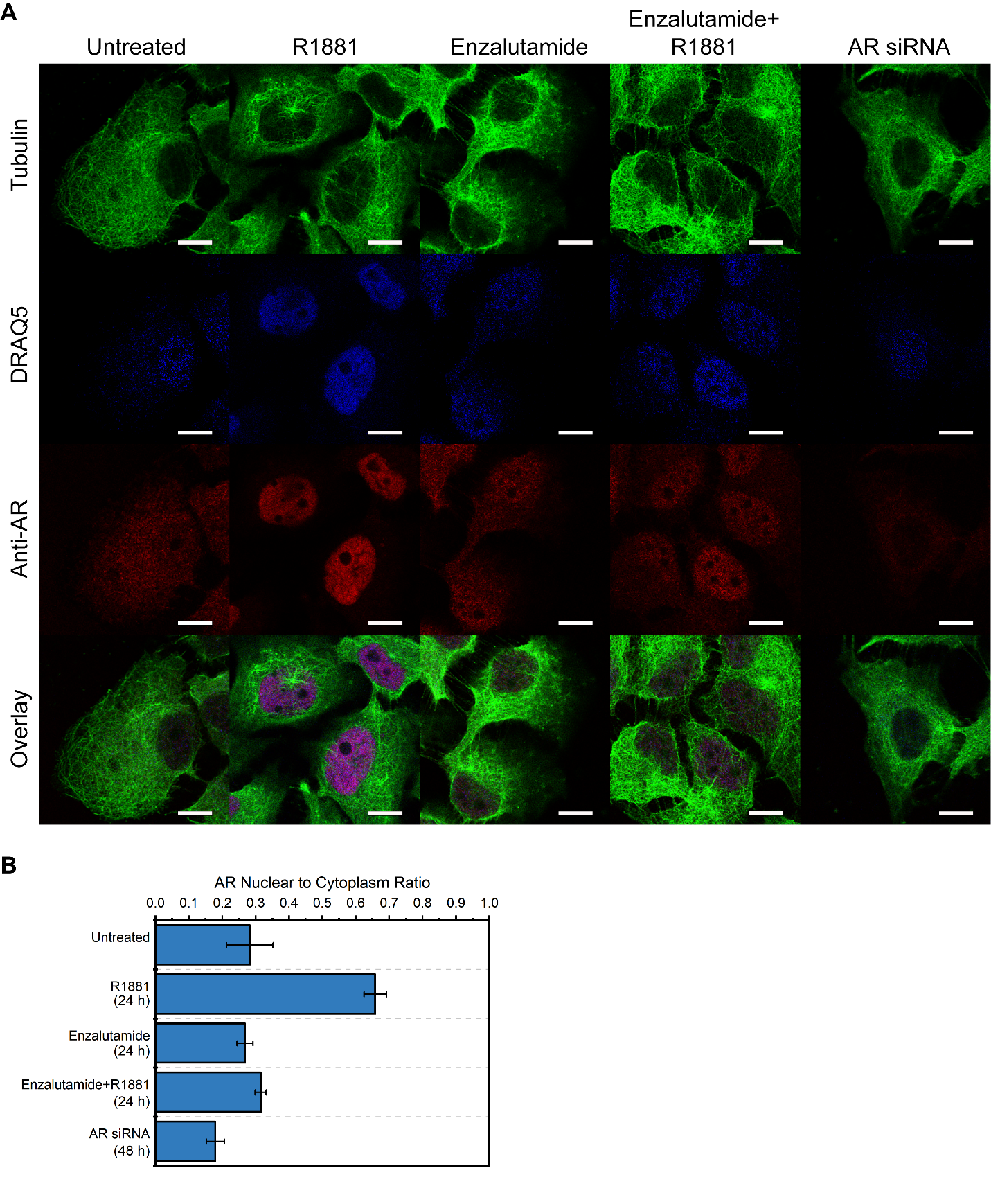


**Supplementary Figure S2.** AR immunofluorescent images for NCI-H2228 cells**. (A)** Representative immunofluorescent images of AR proteins in H2228 treated with 1nM R1881, 5μM Enzalutamide (with or without 1 nM R1881), or AR siRNA (scale bar: 10µm). **(B)** Ratio of quantified AR immunofluorescent signals within the nucleus to the entire cell region (H2228). Data are presented as Mean ± SD.





**Supplementary Figure S3.** AR siRNA transfection optimization. Three different siRNA sequences were tested for effectiveness of AR mRNA degradation. A549 cells were transfected with individual sequences at 10nM concentration using lipofectamine for 24h and 48h. The mRNA samples were extracted, and the AR mRNA levels were measured with qRT-PCR. Data is presented as fold change ± SE.


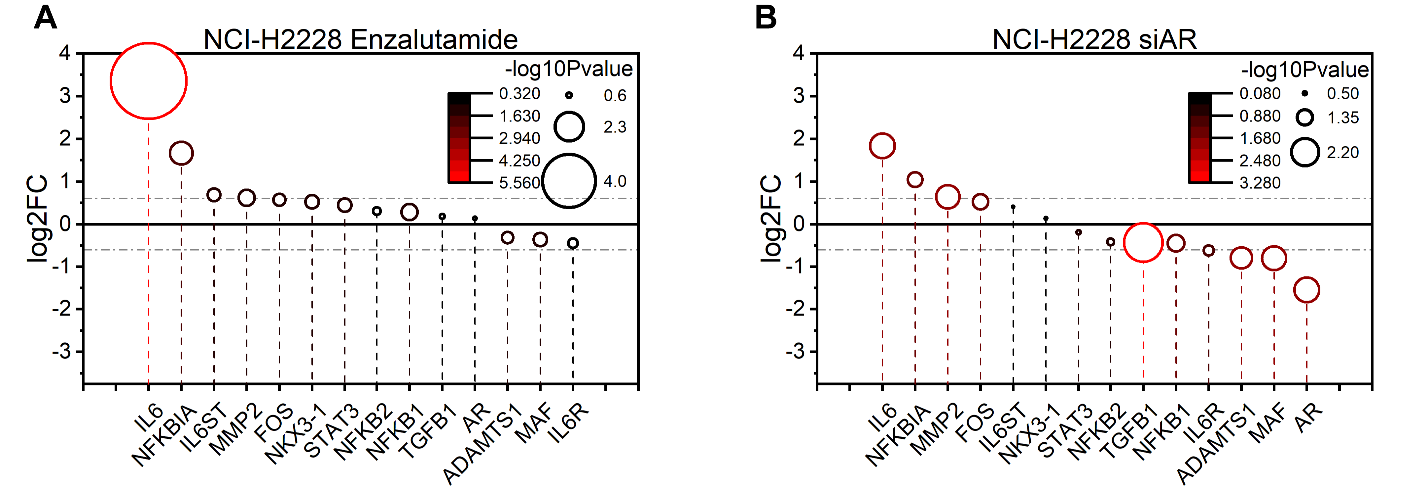


**Supplementary Figure S4.** Log_2_ fold change (log_2_FC) of NCI-H2228 treated with enzalutamide and AR siRNA. In contrast to A549 (Fig. 2E-F), IL6 was significantly upregulated in NCI-H2228. Log_2_FC gene expressions were obtained from AR signaling panels. Genes with absolute values of log_2_FC greater than 0.6 (represented by horizontal grey dash-dotted lines) and p-value less than 0.05 (or 1.3 in -log10 scale) are considered significant. The size and color (black to red) of the circle represent p-values.


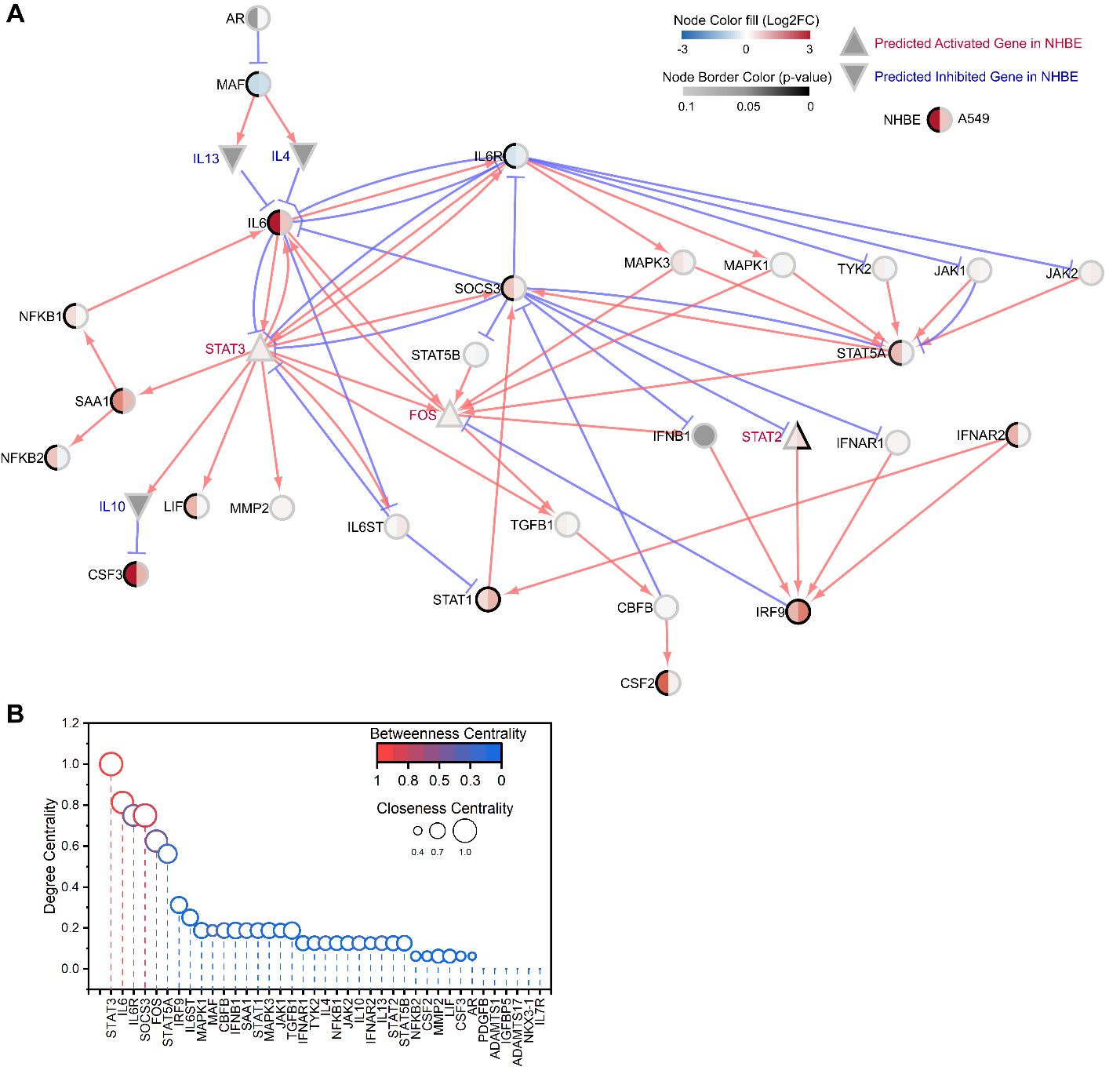


**Supplementary Figure S5.** Full network analysis for NHBE and A549 cells. **(A)** DEGs of NHBE in the JAK-STAT signaling pathway (based on KEGG pathway database) and a custom gene list (see methods for more details) were used to create gene interaction networks. Gene expressions of A549 and NHBE were plotted side by side (left half circle: NHBE, right half circle: A549) for comparison. This is the full version of Figure 1J. **(B)** The degree centrality (y-axis), closeness centrality (circle size), and betweenness centrality (color scale) of each gene in the network are shown here.

###### Supplementary Table S1. EC50 of lung cancer cell lines

| Cell Line | EC50 (µM) | |
| --- | --- | --- |
| A549 | 39.890 | ±2.235 |
| NCI-H2228 | 68.023 | ±2.982 |
| NCI-H226 | 60.362 | ±9.848 |
| NCI-H358 | 66.935 | ±1.714 |
| NCI-H460 | 68.682 | ±14.850 |
| NCI-H520 | 90.046 | ±9.291 |
| NCI-H1299 | 34.204 | ±1.610 |
| NCI-H1650 | 67.067 | ±4.370 |
| NCI-H1975 | 69.471 | ±3.620 |
| NCI-H3122 | 17.318 | ±1.349 |

Half maximal effective concentration (EC50) of enzalutamide treatment on lung cancer cells at 72h. The data are shown as EC50 ± SD.

###### Supplementary Table S2 | AR siRNA sequences

|  | Forward | Reverse |
| --- | --- | --- |
| siAR1 | GUCACAAAGAUUUCUUACCAACUCT | AGAGUUGGUAAGAAAUCUUUGUGACUA |
| siAR2 | CUUUUGACCUGCUAAUCAAGUCACA | UGUGACUUGAUUAGCAGGUCAAAAGUG |
| siAR3 | AUGAAAGCACUGCUACUCUUCAGCA | UGCUGAAGAGUAGCAGUGCUUUCAUGC |

Three different siRNA targeting AR were tested for knockdown efficiency.

###### Supplementary Table S3. Upstream regulator analysis for A549 and NHBE

|  | Genes | | A549 | | | NHBE | | |
| --- | --- | --- | --- | --- | --- | --- | --- | --- |
|  | entity | name | #cDEG | # DEG | FDR | # cDEG | # DEG | FDR |
| Predicted as Activated | 6773 | STAT2 | 14 | 14 | 1.05E-12 | 16 | 16 | 8.18E-13 |
|  | 2353 | FOS | 2 | 2 | 1.66E-01 | 5 | 6 | 2.62E-02 |
|  | 6774 | STAT3 | 4 | 4 | 6.52E-02 | 12 | 16 | 2.10E-03 |
| Predicted as Inhibited | 3565 | IL4 | 2 | 2 | 3.16E-01 | 12 | 16 | 1.08E-04 |
|  | 3596 | IL13 | 1 | 2 | 6.35E-01 | 7 | 10 | 1.14E-02 |
|  |  |  |  |  |  |  |  |  |

A gene from the network is predicted to be activated or inhibited based on the expressions of its downstream differentially expressed (DE) target genes, and the regulatory interactions (activation or inhibition) between it and its DE target genes. The number of DE target genes (# cDEG) that have the sign of their log2 fold changes consistent with the type of regulatory interaction (ex. when the interaction is activation and the target DEG is upregulated, then it increases the likelihood of this upstream regulator is activated), the total number of DEG measured (# DEG), and the false discovery rate (FDR) are shown here.

**Supplementary Table S4. Selective inflammatory and immune related GO Biological Process terms**

|  |  | A549 | | | | NHBE | | | |
| --- | --- | --- | --- | --- | --- | --- | --- | --- | --- |
| name | **GO ID** | **Count DEG** | **Count All** | **% gene** | **p-value (Elim)** | **Count DEG** | **Count All** | **% gene** | **p-value (Elim)** |
| type I interferon signaling pathway | GO:0060337 | 18 | 61 | 29.5 | 5.70E-24 | 18 | 72 | 25.0 | 8.70E-15 |
| defense response to virus | GO:0051607 | 24 | 151 | 15.9 | 2.60E-17 | 23 | 172 | 13.4 | 8.30E-11 |
| negative regulation of viral genome replication | GO:0045071 | 12 | 40 | 30.0 | 7.00E-17 | 12 | 47 | 25.5 | 1.30E-11 |
| interferon-gamma-mediated signaling pathway | GO:0060333 | 10 | 56 | 17.9 | 1.80E-09 | 8 | 64 | 12.5 | 1.10E-04 |
| innate immune response | GO:0045087 | 41 | 483 | 8.5 | 6.00E-08 | 51 | 560 | 9.1 | 5.60E-05 |
| regulation of complement activation | GO:0030449 | 5 | 16 | 31.3 | 3.70E-06 | 6 | 20 | 30.0 | 1.60E-04 |
| complement activation, alternative pathway | GO:0006957 | 3 | 5 | 60.0 | 4.20E-06 | 2 | 3 | 66.7 | 8.60E-04 |
| positive regulation of defense response to virus by host | GO:0002230 | 4 | 17 | 23.5 | 6.80E-06 | 2 | 18 | 11.1 | 3.70E-02 |
| negative regulation of type I interferon production | GO:0032480 | 5 | 36 | 13.9 | 6.90E-06 | 4 | 39 | 10.3 | 4.23E-03 |
| complement activation, classical pathway | GO:0006958 | 3 | 9 | 33.3 | 3.40E-05 | 3 | 14 | 21.4 | 1.55E-03 |
| response to interferon-beta | GO:0035456 | 5 | 17 | 29.4 | 5.20E-04 | 7 | 21 | 33.3 | 3.60E-08 |
| response to interferon-alpha | GO:0035455 | 4 | 15 | 26.7 | 7.90E-04 | 6 | 19 | 31.6 | 5.10E-07 |
| regulation of type I interferon-mediated signaling pathway | GO:0060338 | 3 | 26 | 11.5 | 9.60E-04 | 4 | 27 | 14.8 | 3.27E-02 |
| positive regulation of response to cytokine stimulus | GO:0060760 | 5 | 36 | 13.9 | 1.65E-03 | 6 | 43 | 14.0 | 2.20E-03 |
| positive regulation of interleukin-6 secretion | GO:2000778 | 2 | 14 | 14.3 | 4.88E-03 | 3 | 20 | 15.0 | 4.50E-03 |
| cellular response to interleukin-6 | GO:0071354 | 2 | 22 | 9.1 | 1.19E-02 | 4 | 25 | 16.0 | 1.17E-02 |
| positive regulation of tyrosine phosphorylation of STAT protein | GO:0042531 | 2 | 22 | 9.1 | 1.19E-02 | 6 | 35 | 17.1 | 2.40E-05 |
| cellular response to virus | GO:0098586 | 5 | 39 | 12.8 | 1.76E-02 | 6 | 42 | 14.3 | 2.20E-03 |
| response to virus | GO:0009615 | 26 | 210 | 12.4 | 2.44E-02 | 30 | 239 | 12.6 | 1.40E-04 |
| acute inflammatory response | GO:0002526 | 8 | 58 | 13.8 | 3.04E-02 | 17 | 72 | 23.6 | 5.49E-03 |
| response to interferon-gamma | GO:0034341 | 12 | 106 | 11.3 | 4.33E-02 | 17 | 128 | 13.3 | 2.70E-06 |

The GO terms were ordered by the p-values obtained from A549 cells. The number of differentially expressed genes (Count DEG) was shown with the total number of measured genes (Count All). The % gene was calculated by (Count DEG)/(Count All). Elim method was used to correct p-values.

Supplementary Table S5. Top 20 enriched Hallmark gene sets for A549 and NHBE in GSEA

| A549 | | | | | NHBE | | | | |
| --- | --- | --- | --- | --- | --- | --- | --- | --- | --- |
| NAME | **SIZE** | **ES** | **NES** | **FDR** | **NAME** | **SIZE** | **ES** | **NES** | **FDR** |
| Complement | 166 | 0.489 | 1.766 | 0.081 | **KRAS Signaling Up** | 162 | 0.569 | 2.178 | 0.041 |
| Inflammatory Response | 154 | 0.552 | 1.715 | 0.062 | **Apoptosis** | 153 | 0.452 | 1.965 | 0.041 |
| TNFA Signaling via NFKB | 191 | 0.496 | 1.710 | 0.055 | **IL6 JAK STAT3 Signaling** | 68 | 0.677 | 1.886 | 0.041 |
| Coagulation | 108 | 0.428 | 1.669 | 0.105 | **Complement** | 173 | 0.533 | 1.828 | 0.041 |
| IL6 JAK STAT3 Signaling | 71 | 0.529 | 1.657 | 0.145 | **Interferon Gamma Response** | 183 | 0.656 | 1.812 | 0.041 |
| Interferon Gamma Response | 177 | 0.800 | 1.653 | 0.128 | **Allograft Rejection** | 145 | 0.464 | 1.809 | 0.041 |
| Allograft Rejection | 135 | 0.500 | 1.636 | 0.121 | **TNFA Signaling Via NFKB** | 194 | 0.718 | 1.806 | 0.041 |
| Unfolded Protein Response | 113 | 0.374 | 1.603 | 0.119 | **Inflammatory Response** | 159 | 0.622 | 1.802 | 0.041 |
| KRAS Signaling Up | 162 | 0.448 | 1.533 | 0.135 | **MYC Targets V2** | 58 | 0.560 | 1.734 | 0.041 |
| Interferon Alpha Response | 95 | 0.868 | 1.476 | 0.132 | **Hypoxia** | 187 | 0.379 | 1.716 | 0.041 |
| Apoptosis | 142 | 0.316 | 1.467 | 0.132 | **Unfolded Protein Response** | 112 | 0.421 | 1.690 | 0.052 |
| MYC Targets V1 | 200 | 0.453 | 1.450 | 0.138 | **UV Response Up** | 143 | 0.440 | 1.687 | 0.051 |
| Angiogenesis | 31 | 0.421 | 1.449 | 0.131 | **Interferon Alpha Response** | 97 | 0.721 | 1.683 | 0.050 |
| Glycolysis | 186 | 0.290 | 1.441 | 0.132 | **IL2 STAT5 Signaling** | 172 | 0.376 | 1.653 | 0.053 |
| DNA Repair | 150 | 0.246 | 1.438 | 0.126 | **Reactive Oxygen Species Pathway** | 48 | 0.371 | 1.585 | 0.069 |
| UV Response Up | 149 | 0.259 | 1.416 | 0.133 | **Coagulation** | 114 | 0.444 | 1.553 | 0.070 |
| Hypoxia | 185 | 0.235 | 1.413 | 0.128 | **mTORC1 Signaling** | 198 | 0.284 | 1.549 | 0.071 |
| Epithelial Mesenchymal Transition | 176 | 0.303 | 1.410 | 0.130 | **Cholesterol Homeostasis** | 70 | 0.361 | 1.543 | 0.070 |
| IL2 STAT5 Signaling | 172 | 0.300 | 1.382 | 0.146 | **Xenobiotic Metabolism** | 166 | 0.333 | 1.459 | 0.117 |
| Fatty Acid Metabolism | 141 | 0.305 | 1.354 | 0.175 | **Epithelial Mesenchymal Transition** | 191 | 0.395 | 1.440 | 0.129 |

Top 20 GSEA Hallmark gene sets for A549 (left) and NHBE (right) RNA sequencing data. The size of the gene set, enrichment score (ES), normalized enrichment score (NES), and false discovery rate (FDR) are shown. The gene sets are ordered based on NES of individual cells.

# Supplementary Table S6. Results of network analysis for A549 and NHBE

| Genes | | Centrality | | | A549 | | | NHBE | | |
| --- | --- | --- | --- | --- | --- | --- | --- | --- | --- | --- |
| name | **entrez** | **Degree** | **Closeness** | **Betweenness** | **log2FC** | **p-value** | **adj pv** | **log2FC** | **p-value** | **adj pv** |
| STAT3 | 6774 | 1 | 1 | 1 | 0.04 | 6.42E-01 | 8.92E-01 | 0.19 | 3.30E-02 | 3.37E-01 |
| IL6 | 3569 | 0.813 | 0.947 | 0.929 | 0.97 | 7.69E-02 | NA | 3.05 | 2.17E-23 | 7.96E-21 |
| IL6R | 3570 | 0.750 | 0.926 | 0.381 | -0.28 | 1.28E-01 | 5.42E-01 | -0.58 | 1.17E-05 | 8.54E-04 |
| SOCS3 | 9021 | 0.750 | 1 | 0.810 | 0.22 | 1.23E-01 | 5.35E-01 | 1.04 | 2.74E-07 | 2.76E-05 |
| FOS | 2353 | 0.625 | 0.959 | 0.421 | 0.41 | 1.63E-01 | 5.89E-01 | 0.13 | 1.95E-01 | 6.89E-01 |
| STAT5A | 6776 | 0.563 | 0.829 | 0.166 | -0.16 | 3.79E-01 | 7.53E-01 | 1.18 | 2.77E-04 | 1.23E-02 |
| IRF9 | 10379 | 0.313 | 0.742 | 0.089 | 2.43 | 1.12E-88 | 2.74E-85 | 1.29 | 5.65E-29 | 2.69E-26 |
| IL6ST | 3572 | 0.250 | 0.716 | 0.054 | 0.29 | 2.16E-01 | 6.42E-01 | -0.02 | 8.95E-01 | 9.82E-01 |
| CBFB | 865 | 0.188 | 0.689 | 0.179 | 0.01 | 9.70E-01 | 9.93E-01 | 0.01 | 9.17E-01 | 9.87E-01 |
| IFNB1 | 3456 | 0.188 | 0.714 | 0.006 | NA | NA | NA | NA | NA | NA |
| JAK1 | 3716 | 0.188 | 0.631 | 0.001 | 0.04 | 5.70E-01 | 8.61E-01 | 0.15 | 1.10E-01 | 5.64E-01 |
| MAF | 4094 | 0.188 | 0.533 | 0.216 | -0.68 | 3.11E-01 | NA | -0.83 | 9.05E-09 | 1.20E-06 |
| MAPK1 | 5594 | 0.188 | 0.693 | 0.003 | 0.03 | 7.32E-01 | 9.21E-01 | -0.05 | 5.70E-01 | 9.04E-01 |
| MAPK3 | 5595 | 0.188 | 0.693 | 0.003 | 0.11 | 2.95E-01 | 7.03E-01 | 0.41 | 8.05E-03 | 1.44E-01 |
| SAA1 | 6288 | 0.188 | 0.681 | 0.171 | 1.24 | 1.85E-02 | NA | 2.20 | 2.08E-70 | 5.95E-67 |
| STAT1 | 6772 | 0.188 | 0.689 | 0.110 | 1.32 | 2.21E-51 | 2.71E-48 | 0.54 | 2.39E-08 | 2.97E-06 |
| TGFB1 | 7040 | 0.188 | 0.738 | 0.053 | 0.02 | 8.35E-01 | 9.54E-01 | 0.17 | 1.15E-01 | 5.73E-01 |
| IFNAR1 | 3454 | 0.125 | 0.664 | 0.005 | 0.03 | 8.24E-01 | 9.51E-01 | 0.08 | 4.82E-01 | 8.70E-01 |
| IFNAR2 | 3455 | 0.125 | 0.569 | 0.007 | -0.01 | 9.51E-01 | 9.87E-01 | 1.38 | 3.10E-05 | 1.92E-03 |
| IL10 | 3586 | 0.125 | 0.648 | 0.146 | NA | NA | NA | NA | NA | NA |
| IL13 | 3596 | 0.125 | 0.642 | 0.136 | NA | NA | NA | NA | NA | NA |
| IL4 | 3565 | 0.125 | 0.642 | 0.136 | NA | NA | NA | NA | NA | NA |
| JAK2 | 3717 | 0.125 | 0.631 | 0.001 | 0.24 | 2.80E-01 | 6.94E-01 | 0.15 | 4.28E-01 | 8.49E-01 |
| NFKB1 | 4790 | 0.125 | 0.642 | 0.023 | 0.00 | 9.75E-01 | 9.93E-01 | 0.54 | 8.86E-08 | 9.67E-06 |
| STAT2 | 6773 | 0.125 | 0.664 | 0.005 | 0.59 | 3.59E-08 | 6.76E-06 | 0.28 | 3.36E-03 | 8.23E-02 |
| STAT5B | 6777 | 0.125 | 0.681 | 0.002 | -0.08 | 4.09E-01 | 7.73E-01 | 0.01 | 9.55E-01 | 9.93E-01 |
| TYK2 | 7297 | 0.125 | 0.631 | 0.001 | -0.09 | 4.09E-01 | 7.73E-01 | 0.20 | 1.42E-01 | 6.16E-01 |
| AR | 367 | 0.063 | 0.4 | 0 | -0.04 | 7.05E-01 | 9.13E-01 | -0.87 | 4.98E-01 | NA |
| CSF2 | 1437 | 0.063 | 0.486 | 0 | 0.17 | 7.19E-01 | NA | 2.93 | 6.93E-10 | 1.12E-07 |
| CSF3 | 1440 | 0.063 | 0.470 | 0 | 1.35 | 1.07E-01 | NA | 5.03 | 2.26E-20 | 7.16E-18 |
| LIF | 3976 | 0.063 | 0.615 | 0 | 0.02 | 8.49E-01 | 9.58E-01 | 1.31 | 7.66E-31 | 4.05E-28 |
| MMP2 | 4313 | 0.063 | 0.615 | 0 | 0.08 | 7.11E-01 | 9.14E-01 | 0.08 | 5.19E-01 | 8.86E-01 |
| NFKB2 | 4791 | 0.063 | 0.482 | 0 | -0.16 | 1.08E-01 | 5.15E-01 | 1.01 | 6.47E-20 | 1.97E-17 |
| ADAMTS1 | 9510 | 0 | 0 | 0 | -0.49 | 7.73E-01 | NA | 0.14 | 1.42E-01 | 6.16E-01 |
| ADAMTS17 | 170691 | 0 | 0 | 0 | NA | NA | NA | 0.38 | 8.29E-01 | NA |
| IGFBP5 | 3488 | 0 | 0 | 0 | 0.21 | 6.94E-01 | NA | -1.61 | 4.68E-01 | NA |
| IL7R | 3575 | 0 | 0 | 0 | 3.09 | 8.86E-02 | NA | 0.84 | 7.17E-04 | 2.63E-02 |
| NKX3-1 | 4824 | 0 | 0 | 0 | 0.06 | 7.24E-01 | 9.20E-01 | 0.54 | 4.62E-02 | 3.91E-01 |
| PDGFB | 5155 | 0 | 0 | 0 | -0.21 | 1.68E-01 | 5.93E-01 | 1.01 | 1.71E-08 | 2.17E-06 |

Data for all the nodes in the network analysis (Fig. 1J and Fig. S5A) are shown here. The list is ranked by degree centrality.
